# Energy Component Analysis for Electronically Excited States of Molecules: Why the Lowest Excited State Is Not Always the HOMO/LUMO Transition

**DOI:** 10.1021/acs.jctc.3c00125

**Published:** 2023-04-06

**Authors:** Patrick Kimber, Felix Plasser

**Affiliations:** Department of Chemistry, Loughborough University, Loughborough LE11 3TU, United Kingdom

## Abstract

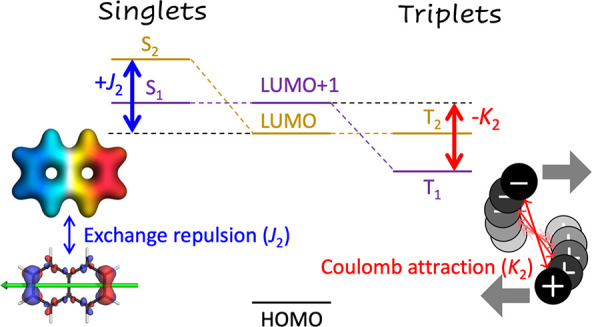

The ability to tune excited-state energies is crucial to many areas of molecular design. In many cases, this is done based on the energies of the highest occupied molecular orbital (HOMO) and lowest unoccupied molecular orbital (LUMO). However, this viewpoint is incomplete neglecting the many-body nature of the underlying excited-state wave functions. Within this work, we highlight the importance of two crucial terms, other than orbital energies, that contribute to the excitation energies and show how to quantify them from quantum chemistry computations: a Coulomb attraction and a repulsive exchange interaction. Using this framework, we explain under which circumstances the lowest excited state of a molecule, of either singlet or triplet multiplicity, is not accessed via the HOMO/LUMO transition and show two paradigmatic examples. In the case of the push–pull molecule **ACRFLCN**, we highlight how the lowest triplet excited state is a locally excited state lying below the HOMO/LUMO charge transfer state due to enhanced Coulomb binding. In the case of the naphthalene molecule, we highlight how the HOMO/LUMO transition (the ^1^*L*_*a*_ state) becomes the second excited singlet state due to its enhanced exchange repulsion term. More generally, we explain why excitation energies do not always behave like orbital energy gaps, providing insight into photophysical processes as well as methodogical challenges in describing them.

## 1 Introduction

The ability to tune electronic excitation energies of molecules is crucial in the development of materials for optoelectronic applications, and particularly, the relative energies of the first singlet and triplet excited states are prominent parameters of high current interest.^1−3^ The design of new molecules is usually based on the energies and shapes of the highest occupied molecular orbital (HOMO) and the lowest unoccupied molecular orbital (LUMO) of the molecules studied. This viewpoint provides a useful starting point, e.g., when estimating the expected effect of different substituents. However, the HOMO/LUMO picture does not tell the whole story,^4,5^ and there are many molecules where the HOMO/LUMO transition does not constitute the lowest excited state of a given spin multiplicity. The HOMO and LUMO energies obtained can be strongly method dependent.^6,7^ More specifically, it is challenging to rationalize singlet–triplet gaps from viewing the HOMO and LUMO independently. Considering, thus, the wide ranging applications but also shortcomings of the HOMO/LUMO picture, it could be highly beneficial to have a systematic way to go beyond it. In particular, the availability of a scheme for decomposing excitation energies into physically motivated energy components could have great potential.

For ground state molecules, a variety of popular energy decomposition analysis schemes are available.^8−10^ These provide a quantitative decomposition of the computed interaction energies for bonding and noncovalent interactions into different physically motivated energy components, e.g., Coulomb, exchange, and polarization, providing detailed insight into the interactions studied. By contrast, much of the focus in the analysis of excited state computations is on a qualitative characterization of state character, for example, in terms of charge transfer,^11−14^ double excitations,^15,16,100^ and multiconfigurational character.^17^ The tuning of excitation energies, on the other hand, usually proceeds via qualitative rules of thumb with no quantitative counterpart, e.g., regarding electron donating/withdrawing groups or delocalization and—for singlet/triplet gaps—in terms of HOMO/LUMO overlap,^18^ radical character,^19^ or excited-state aromaticity.^20^ More quantitative approaches to decomposing excitation energies^21,22^ have not reached maturity for routine use. This is arguably because the physical meaning of the terms involved is not *a priori* clear, no graphical representations are available, and the benefits of such methods to routine problems have not been highlighted.

It is the purpose of this work to present a clear physical picture for decomposing excitation energies into meaningful energy components combined with an intuitive graphical representation and to use this scheme to explain the breakdown of the MO picture. Using preliminary ideas by us and co-workers^22−24^ we develop a consistent and physically meaningful framework providing well-defined excitation energy components for quantum chemistry computations. Building on the MO picture, we provide a clear set of correction terms that bridge between the MO energy gap and the full excitation energy. The energy terms are readily connected with a graphical analysis scheme presented previously.^24^ We use the developed tools to highlight under what circumstances the lowest excited state (of either singlet or triplet spin multiplicity) of a molecule is not derived from the HOMO/LUMO transition. More generally, we highlight the influence of an attractive Coulomb term and a repulsive exchange term on the state ordering and relative energies.

**Figure 1:**
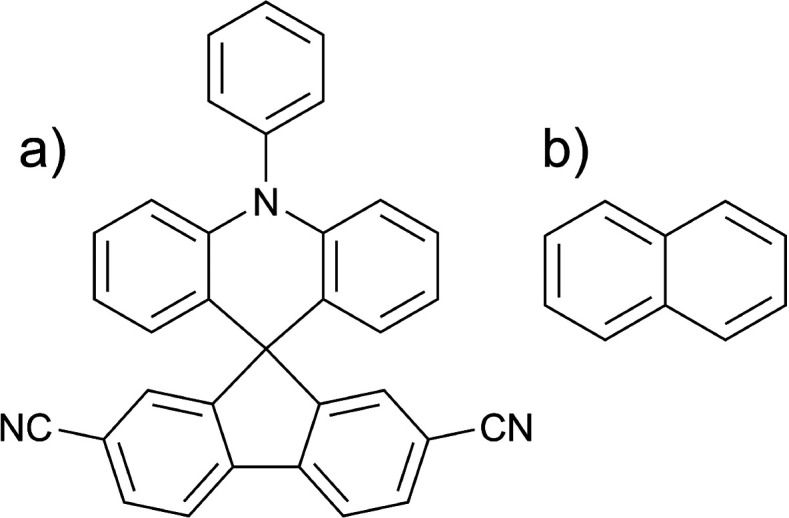
Molecular structures of (a) ACRFLCN and (b) naphthalene.

Two paradigmatic examples are chosen to illustrate the energy component analysis and, in particular, to highlight the breakdown of the HOMO/LUMO picture in systems of practical interest: the push–pull molecule **ACRFLCN** (10-phenyl-10H-spiro[acridine-9,9′-fluorene]-2′,7′-dicarbonitrile) and the naphthalene molecule (Figure 1). The **ACRFLCN** molecule^25^ is chosen to represent the class of donor–acceptor systems with a low singlet–triplet gap employed in thermally activated delayed fluorescence (TADF). Within TADF emitters, the interplay of different locally excited (LE) and charge transfer (CT) states is particularly important where the CT character is decisive for lowering the singlet–triplet gap, whereas LE character promotes spin–orbit coupling.^26^ Below, we investigate the different energy components determining the relative stability of these LE and CT states. Naphthalene, on the other hand, represents the case of a typical aromatic molecule where quasidegeneracies in the π-system lead to a set of ππ* states of quite distinct character despite involving similar orbital transitions.^27−29^ In this case, we illustrate how our energy component analysis casts new light onto the differences between the ionic “+” and covalent “–” states present in these systems.^30,31^ We explain the consequences on their spectroscopic properties as well as on their computational description with ab initio methods.^32,33^ Below, we show that the lowest singlet state (*S*_1_) of **ACRFLCN** is indeed the HOMO/LUMO transition, whereas the triplet (*T*_1_) is not. Naphthalene represents the opposite case where *T*_1_ is dominated by the HOMO/LUMO transition, but *S*_1_ is not.

## 2 Methods

Within this section, we start with a general discussion of a physically motivated energy decomposition proceeding from a two orbital model to general ab initio computations. We continue with a more technical part outlining the detailed energy terms involved in range-separated hybrid time-dependent density functional theory (TDDFT). Subsequently, a more general interpretation of the significance of the derived energy terms is presented. We conclude with the computational details. Within this section, we aim for a compact and intuitive explanation of the terms involved, while a more detailed derivation is presented in ref (24).

### 2.1 Excited-State Energy Decomposition

**Figure 2:**
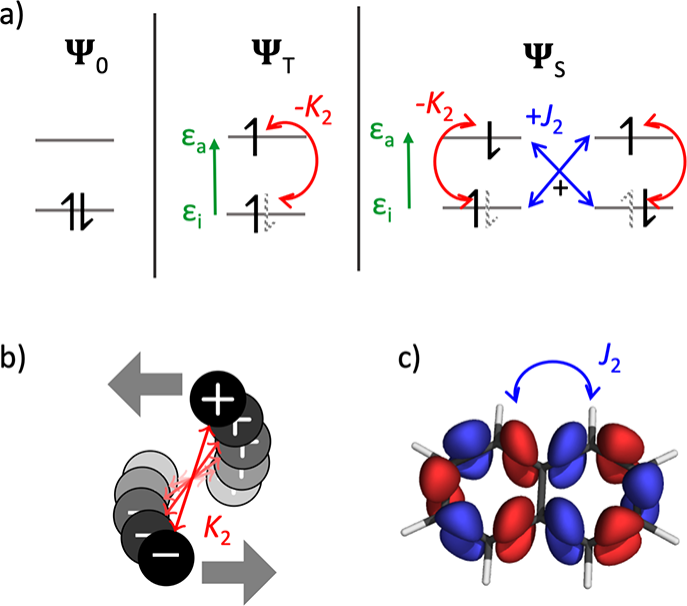
Depiction of energy terms contributing to excited-state energies: (a) excitation energies of triplet (Ψ_*T*_) and singlet (Ψ_*S*_) excited states within a two-orbital two-electron model; generalization of (b) the *K*_2_ term as a dynamic electron–hole binding energy, and (c) the *J*_2_ term as a transition density repulsion.

We use a two-orbital two-electron model (TOTEM)^24,34,35^ as a conceptual starting point to illustrate the physics involved and proceed to a more general discussion later. The singlet and triplet excitation energies within the TOTEM are presented in Figure 2. The first component, applicable to singlets and triplets alike, is the energy required to promote the electron from the occupied orbital ϕ_*i*_ to the virtual orbital ϕ_*a*_. It as represented by the orbital energy gap ϵ_*a*_ – ϵ_*i*_.

However, the orbital energy gap is not generally a good guess for the excitation energy. To explain this, we start with the simplest possible construction of an excited state, a triplet state formed as a single Slater determinant; an electron is promoted from ϕ_*i*_ and to ϕ_*a*_ with its spin flipped. To obtain the energy of this Slater determinant, we first have to remove the electron from ϕ_*i*_. According to Koopmans’ theorem, this requires an energy of ϵ_*i*_, given as
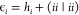
1where *h*_*i*_ is the one-electron term, and (*ii*|*ii*) is the Coulomb repulsion between the two-electrons located in orbital ϕ_*i*_. To reattach the electron, we can also apply Koopmans’ theorem using the orbital energy of ϕ_*a*_ as an estimate of the electron affinity. However, the crucial realization is that we have to use the orbital energy evaluated with respect to the charged system
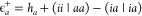
2rather than the orbital energy in the neutral system
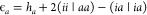
3which differ by one Coulomb term (*ii*|*aa*). In summary, the energy of a triplet state in the TOTEM is
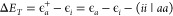
4

The energy is shifted by the Coulomb attraction (*ii*|*aa*) between occupied and virtual orbitals, a term that we generally denote as *K*_2_. The name *K*_2_ is chosen as this term derives from the response of the exchange potential as discussed in more detail below. *K*_2_ can be interpreted in the sense that the excited electron is attracted by the *hole* it left in the ground-state density (indicated as a dashed line in Figure 2).

Open-shell singlet excited states are generally constructed from at least two Slater determinants. Hence, the derivation of the energy terms is slightly more involved;^24,36^ one obtains the expression
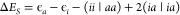
5containing the *K*_2_ term and an additional term 2(*ia*|*ia*) that we generally denote as *J*_2_. The *J*_2_ term only applies to singlet states. It is generally repulsive and interpreted as an exchange interaction between electron and hole.

To turn the above consideration into a practically useful tool, it is worth realizing that the energies of most excited-state quantum chemistry methods naturally comply with the above decomposition. Taking configuration interaction singles (CIS) or TDDFT in the Tamm–Dancoff approximation (TDA) as an example,^6,24^ the excitation energies can be written in the following way
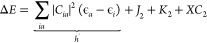
6Here, the *C*_*ia*_ are the TDDFT response coefficients, and *J*_2_/*K*_2_ are the two-electron terms deriving from the response of the Coulomb and nonlocal exchange potentials. The precise forms of these terms are discussed below, but for now, it is enough to realize that they are natural generalizations of the (*ia*|*ia*) and (*ii*|*aa*) terms mentioned above. The *XC*_2_ term contains any additional contributions deriving from exchange or correlation effects. Within TDDFT, the *XC*_2_ term is explicitly evaluated as the response of the exchange-correlation potential. Within CIS, the *XC*_2_ term vanishes. However, it would be a natural generalization within a correlated ab initio framework to define *XC*_2_ as the energy difference between CIS and the correlated method used.

Furthermore, the one-electron term, denoted *h*′, is given as a weighted sum of orbital energy differences as in eq 6. It can be written explicitly as
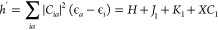
7containing the one-electron core Hamiltonian (*H*) and one-electron terms *J*_1_, *K*_1_; *XC*_1_ is computed from the ground-state Fock matrix. However, we do not use this decomposition in the following but consider the *h*′ term as a whole.

All terms in eqs 6 and 7 can be readily obtained from CIS or hybrid TDDFT/TDA computations as reported previously^22,23^ providing an interesting practical basis for our analysis.

### 2.2 Practical Evaluation

Equation 6 provides a well-defined decomposition of excitation energies in terms of the MO energy gaps and three additional two-electron terms. However, there is some arbitrariness in terms of how to compute the individual terms. From a physical point of view, one can study why the excitation energy is not equivalent to a difference in electron affinity and ionization potential and what other terms contribute. From a more applied point of view, one can ask if, for any given method, MO energy gaps provide a good guess to the excitation energies. In this context, it should be noted that orbital energies can be strongly method dependent differing between Hartree–Fock and the various density functionals. Within Hartree–Fock, the HOMO/LUMO gap represents—at least in principle—the fundamental gap via Koopmans’ theorem. By contrast, for Kohn–Sham DFT with local functionals (e.g., BLYP, PBE) or global hybrid functionals with not too much exchange (e.g., B3LYP, PBE0), the HOMO/LUMO gap is already a good guess for the excitation energy noting that occupied and virtual orbitals experience the same Kohn–Sham potential.^6,7,37^ Within the presented formalism, the difference is encoded in a balance between the *K*_1_ and *K*_2_ terms. Enhanced nonlocal exchange increases the HOMO/LUMO gap via an increased *K*_1_ term, while this is compensated for by the excitation energy via an enhanced *K*_2_ term. As a consequence, the excitation energies for locally excited states are fairly insensitive to the amount of nonlocal exchange despite dramatic changes in the HOMO/LUMO gap.

Within this work, we use TDDFT with an optimally tuned range-separated density functional. This is aimed at combining both viewpoints just described, since the obtained HOMO and LUMO energies are *per construction* good guesses of the ionization potential and electron affinity.^38^

Within TDDFT/TDA and CIS, the *J*_2_ term of eq 6 is evaluated as a linear combination of exchange-type two-electron integrals
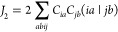
8where *C*_*ia*_ are the TDDFT response coefficients (summed over α and β spins). To obtain a more intuitive form of this equation, it is first convenient to introduce the one-electron transition density matrix
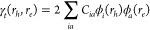
9which is interpreted as the wave function of the electron–hole pair, described by the coordinates *r*_*e*_ and *r*_*h*_.^14,24^ Subsequently, the transition density (ρ_*t*_) is defined as ρ_*t*_(*r*) = γ_*t*_(*r*, *r*). Using this definition, eq 8 is rewritten as^24^
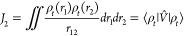
10where *r*_12_ is the interelectronic separation. *J*_2_ is simply the Coulomb repulsion of the transition density with respect to itself, as illustrated in Figure 2(c). As shown on the right hand side of eq 10, the *J*_2_ term can be viewed as the overlap of the transition density (ρ_*t*_) with the electrostatic potential (ESP) induced by it (*Vρ̂*_*t*_), and we suggest using this for a graphical representation (see below).^24^

The *K*_2_ term within range-separated TDA–TDDFT is slightly more involved. It can be written as a linear combination of modified two-electron integrals (cf. refs (6, 39))
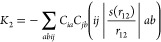
11where we use *s*(*r*_12_) to denote the function used for the range separation procedure. Within the LRC-ωPBEh functional^40^ employed here, this function is defined as
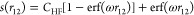
12where *C*_HF_ is the global fraction of Hartree–Fock exchange, ω is the range-separation parameter, and erf denotes the error function. Note that for a global hybrid functional, one would simply set *s*(*r*_12_) = *C*_HF_, and the analogous term for configuration interaction singles (CIS) is obtained with *s*(*r*_12_) = 1.

Using eq 9, *K*_2_ is re-expressed in the form
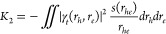
13Thus, *K*_2_ can be seen as a screened Coulomb interaction between the electron and hole quasiparticles where the screening is determined by the function *s*(*r*_12_).

Equation 13 highlights that *K*_2_ is generally a correlated term depending in a nontrivial fashion on the two-body function γ_*t*_(*r*_*h*_, *r*_*e*_) describing the mutual distribution of *electron* and *hole*; this dynamic nature is represented graphically in Figure 2(b). Conversely, if no correlation is present and γ_*t*_ can be factorized into a single pair of natural transition orbitals (NTOs), then *K*_2_ takes on the simpler form of a (screened) Coulomb interaction between the *hole* (ρ_*h*_) and *electron* (ρ_*e*_) densities^24^
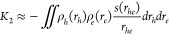
14Under this approximation, the *K*_2_ term can be represented via plots of ρ_*h*_/ρ_*e*_ and their ESPs in analogy to eq 10. But it should be remembered that this works only if γ_*t*_ can indeed be represented by only two NTOs (see refs (17, 24) for further discussion).

The last term contributing to the TDA–TDDFT energy is *XC*_2_, the response of the exchange-correlation potential. It is given in analogy to eq 8 only that the electron repulsion integrals are replaced with two-electron integrals (*ia*|*f*_*xc*_|*jb*) involving the response of the exchange-correlation potential^6^
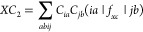
15There is no simple interpretation for the *XC*_2_ term; it is a collection of all contributions to the excitation energy that are not related to the orbital gap, Coulomb attraction, or exchange repulsion. As shown below, within the presented approach, *XC*_2_ represents only a small correction term not affecting the qualitative discussions.

### 2.3 Interpretation of the Energy Terms

A summary of the four relevant terms is presented in Table 1 discussing their origin and their physical interpretation. *h*′ is the weighted MO energy difference and represents the energy required for independent ionization and electron attachment. This term is routinely invoked when explaining excited state energies based on MO energy gaps. Three two-electron terms follow describing the interactions between electron and hole.

**Table 1:** Descriptions of the Four Terms Used in the Energy Decomposition Analysis: Their Origin and Their Physical Interpretation

Term	Origin	Physical interpretation
*h*′	MO energies	Independent ionization and electron attachment
*J*_2_	Coulomb operator	Exchange repulsion between electron and hole
*K*_2_	Exchange operator	Screened Coulomb binding between electron and hole
*XC*_2_	XC functional	Further exchange or correlation effects

*J*_2_ signifies the exchange repulsion between the two electrons present in open-shell singlet states; it vanishes for triplet states (assuming a singlet ground state) considering that also the transition density ρ_*t*_ vanishes in space. *J*_2_ is the dominant term inducing the splitting between singlet and triplet states. Its value is large if the *electron* and *hole* orbitals overlap in space. The exchange repulsion determines the magnitude of the gap between the first singlet and triplet excited states, a crucial quantity for a number of applications, e.g., thermally activated delayed fluorescence, singlet fission, or triplet upconversion.^1−3^

The *K*_2_ term corresponds to a binding energy of the electron–hole pair. It is generally a stabilizing term representing the dynamic Coulomb attraction between the *electron* and *hole* quasiparticles. This Coulomb attraction is the primary contribution to the exciton binding energy, that is, the energy required to separate the *electron*–*hole* pair into free charge carriers, which plays an important role in the operation of solar cells.^41,42^ As explained in more detail above, *K*_2_ is generally a correlated term depending on the two-body distribution of electron and hole. It is by virtue of this term that TDDFT and CIS can describe certain kinds of nondynamic correlation, present in dynamic exciton binding^43^ and charge-resonance effects.^44^

Contrasting *J*_2_ and *K*_2_, it is interesting to note that the response of the Coulomb operator *J*_2_ acts as an exchange-like term on the *electron*–*hole* pair and vice versa for the *K*_2_ term inducing Coulomb binding (see also ref (6)). This induces some potential for confusion in terms of the definition of the terms; for the present purposes, we choose to use the *J* and *K* labels according to the ground-state operators rather than the *electron*–*hole* picture. Furthermore, the presented formalism suggests that *J*_2_ should be computed with an unmodified or “bare” Coulomb interaction, whereas *K*_2_ uses a screened Coulomb interaction as defined by the range-separation procedure. Note that this distinction between the bare exchange and screened Coulomb interaction is also common in the solid-state physics community where the latter is the eponymous *W* term of the GW method.^45,46^

From a technical point of view, it is worth noting that *K*_2_ is the critical term in the TDDFT description of charge transfer states^47^ and delocalized excitons,^43^ where the question of just how much the Coulomb interaction is screened (via *C*_*HF*_ and ω) is decisive on how well such states are described. Conversely, an accurate description of the *J*_2_ term needs to include σ correlation,^24^ as discussed in more detail below. This fact underlies the long-known challenges^48−51^ of describing ionic ππ* states with π-only correlation models.

### 2.4 Computational Details

Vertical excitations for naphthalene and **ACRFCLN** were computed using LRC-ωPBEh/def2-SV(P)^40,52,53^ with 20% global Hartree–Fock exchange (*C*_HF_ = 0.20). Optimally tuned values of ω = 0.140 au for **ACRFLCN** and ω = 0.225 au for naphthalene were used. Here, a tuning procedure was employed that matches the HOMO (ϵ_*H*_) and LUMO (ϵ_*L*_) energies of the neutral system most closely to ionization potential (IP) and electron affinity (EA), respectively. Values obtained for **ACRFLCN** were ϵ_*H*_ = −7.108 eV, IP = 7.063 eV, ϵ_*L*_ = −1.132 eV, and EA = −1.042 e, and for naphthalene were ϵ_*H*_ = −8.163 eV, IP = 8.176 eV, ϵ_*L*_ = 0.544 eV, and EA = 0.545 eV. The geometries were obtained at the same level of theory using the original parameters for LRC-ωPBEh (ω = 0.200 au, *C*_HF_ = 0.20). No solvent model was employed.

Analyses were performed as described above. In addition, the exciton size *d*_*exc*_ defined as a root-mean-square electron–hole separation was computed^14^
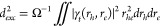
16where Ω is a normalization factor whose value is 1 in the case of TDA–TDDFT. The *d*_*exc*_ value has been shown to be a versatile measure for charge transfer,^14^ exciton binding,^43^ and Rydberg character.^54^ We study its relation to the *K*_2_ term here noting the similarity of eq 16 to eq 13.

In addition, we compute a descriptor, LOC, measuring the probability that *electron* and *hole* are located on the same basis function simultaneously. It is computed as
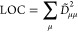
17where *D̃*_*μμ*_ are the diagonal elements of the transition density matrix expressed within the Löwdin-orthogonalized atomic orbital basis. LOC is related to the previously defined fragment-based CT value^44^ where LOC is equivalent to 1 – CT with each basis function seen as its own fragment. As shown below states with LOC = 0.000 are associated with “–” states in Pariser’s nomenclature whereas LOC > 0.03 indicates “+” states.

Calculations were performed using a developmental version of Q-Chem 5.2/6.0.^55^ The libwfa library in Q-Chem is used for wave function analysis, ESPs, and for computing the LOC descriptor.^56^ For technical reasons, ESPs were always computed with an unscreend Coulomb interaction [i.e., *s*(*r*_*he*_) = 1 in eq 14]. Excited state energy component analysis is performed by specifying EXCIT_ENERGY_COMPONENTS = TRUE in the Q-Chem input files.^22^ The TheoDORE toolkit is used for general postprocessing of excited state computations.^57^ PyMOL is used with the external qc_pymol toolkit for plotting ESPs.^58,59^ Q-Chem/TheoDORE input/output files are provided via a separate repository.^60^

## 3 Results and Discussion

Armed with the above toolkit we now want to tackle an intriguing question: When is the lowest state not the HOMO/LUMO transition? The first term in eq 6 is per construction minimized by the HOMO/LUMO transition, since it directly depends on the MO energies. Conversely, the three two-electron terms are responsible for the fact that excitation energies are not equal to orbital energy gaps, and if they differ enough for different types of states, then the HOMO/LUMO transition can be pushed above another state. Two cases can be envisaged. If the stabilizing *K*_2_ term of the HOMO/LUMO transition is small, then another state with larger *K*_2_ may be moved below it. This case is particularly relevant to donor–acceptor systems, where the *K*_2_ terms for locally excited (LE) states are expected to be significantly larger than for charge transfer (CT) states. Specifically triplet LE states (^3^LE) are stabilized this way as they are unaffected by exchange repulsion. Conversely, if the HOMO/LUMO overlap is large, then the *J*_2_ term associated with the singlet HOMO/LUMO transition may dominate, pushing it above another singlet excited state. In principle, also the *XC*_2_ term could dominate. However, we find that it generally only has a minor contribution in the examples presented here.

In summary, the discussion suggests that the post-MO terms are particularly important for LE states where triplet LE states are stabilized via *K*_2_ and singlet LE states are destabilized via *J*_2_. The **ACRFLCN** and naphthalene molecules (see Figure 1) are chosen to exemplify these two cases. We discuss these molecules using the canonical MO picture, the energy component analysis described above, and a pictorial analysis scheme.

### 3.1 **ACRFLCN**: CT and LE States

**Figure 3:**
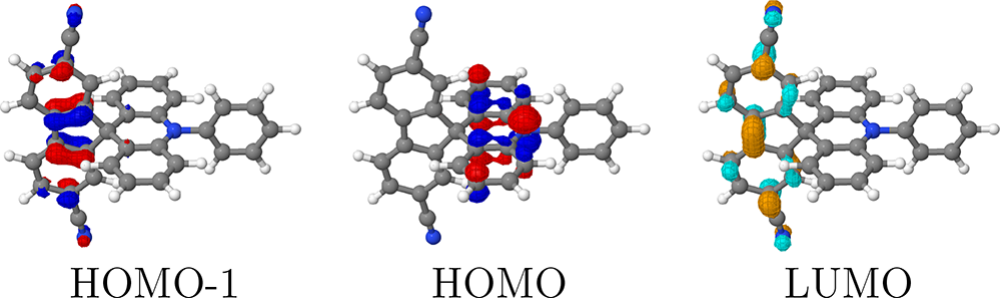
HOMO–1, HOMO, and LUMO for **ACRFLCN** computed at the LRC-ωPBEh/def2-SV(P) level of theory.

**ACRFLCN** is a bichromophoric molecule composed of an acridine donor and a triphenylamine acceptor. The chromophores are connected by a spiro junction forcing them into a perpendicular conformation reducing conformational flexibility and, thus, complexity providing a convenient example for this study. The frontier orbitals of **ACRFLCN** are shown in Figure 3. The HOMO is located on the donor unit (right), whereas the LUMO, along with the HOMO–1 is on the acceptor unit (left). The HOMO and LUMO are associated with independent electron detachment and attachment processes. In line with expectations, Figure 3, thus, indicates that electron detachment involved in the oxidation of **ACRFLCN** would occur on the donor unit, whereas electron attachment (reduction) occurs on the acceptor. Accordingly, the HOMO/LUMO transition is classified as an intramolecular CT state. By contrast, the HOMO–1 is located on the acceptor, and the HOMO–1/LUMO transition is, thus, classified as LE.

TDDFT computations are now performed to study how the orbitals just described are involved in the lowest singlet and triplet states (see Table 2). The HOMO/LUMO gap, computed at the LRC-ωPBEh level, is 5.856 eV, whereas the gap between HOMO–1 and LUMO is 6.933 eV. Nonetheless, we find that the lowest excited state overall, the *T*_1_ state, located at 3.030 eV, is reached predominantly via the HOMO–1 to LUMO transition forming a locally excited state, denoted henceforth as ^3^LE. It is followed by *T*_2_ (^3^CT) at 3.324 eV which is essentially a pure HOMO/LUMO transition. The *S*_1_ (^1^CT) state is found just 0.012 eV higher in energy than *T*_2_ with an almost identical electron configuration to the *T*_2_ state. *S*_1_ is a dark state with negligible oscillator strength. Finally, *S*_2_ (^1^LE) is a bright locally excited state at 4.258 eV and is reached via the HOMO–1/LUMO transition.

**Table 2:** Excitation Energies (Δ*E*, eV), Oscillator Strengths (f), and Leading Configurations for the First Two Singlet and Triplet States of **ACRFLCN** Computed at the LRC-ωPBEh/def2-SV(P) (TDA) Level of theory

State	Δ*E*	f	Configurations^a^
^3^LE (*T*_1_)	3.030	–	0.85*h*_1_*l* + 0.37*h*_3_*l*
^3^CT (*T*_2_)	3.324	–	0.99*hl*
^1^CT (*S*_1_)	3.336	0.000	0.99*hl*
^1^LE (*S*_2_)	4.258	0.334	0.91*h*_1_*l*

a Dominant configurations and coefficients; *h*_*x*_*l*_*y*_ refers to the HOMO-*x*/LUMO+*y* transition.

Before continuing, we note that the energetic difference between *S*_1_ and *T*_1_ as mentioned above is 0.306 eV, somewhat higher than the reported experimental gap of ≈0.1 eV.^25^ The reason for this discrepancy is that the presented values are vertical excitation energies in the gas phase, whereas the experiment considers structurally relaxed molecules in solution. We have studied the influence of structural relaxation and solvation on related systems in detail^61^ but want to omit such a discussion here to focus on the presented decomposition for vertical gas-phase excitation energies. In the future, it is certainly possible to extend the presented analysis scheme to include contributions from solvation and structural relaxation.

Based on the data presented, we highlight two key points: first, the lowest excited states of each multiplicity are of different characters (^3^LE and ^1^CT), where only the singlet is formed via the HOMO/LUMO transition. Second, the energy difference between the ^1^LE and ^3^LE states (1.228 eV) is significantly larger than that between the ^1^CT and ^3^CT states (0.012 eV).

**Figure 4:**
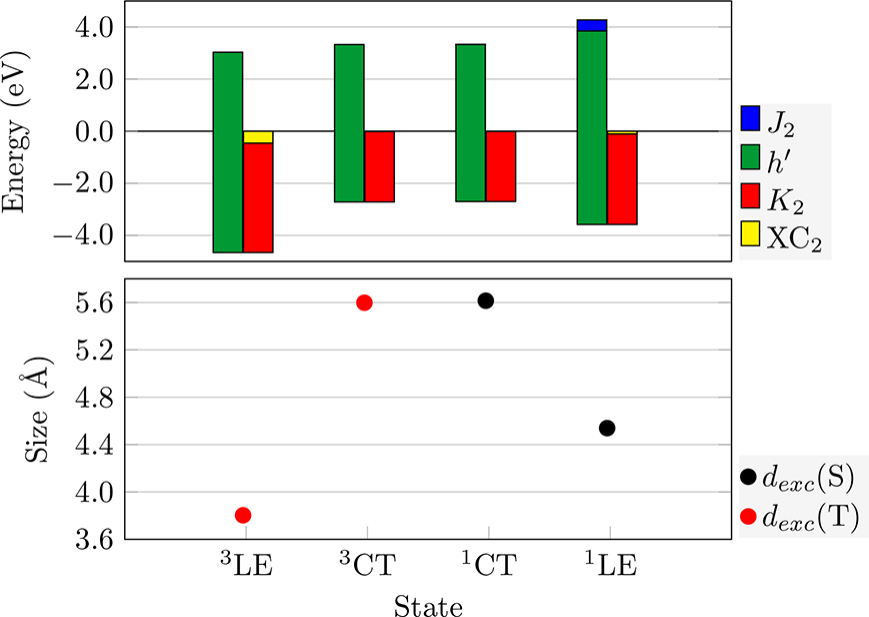
Energy components (top) and exciton size descriptors (bottom) for the computed excited states of **ACRFLCN**. Exciton sizes *d*_*exc*_ are plotted with singlets in black and triplets in red.

To rationalize these findings, we proceed to the energy component analysis presented above. To develop an intuitive graphical representation for the energy contributions, we first realize that the one-electron term *h*′ and the exchange repulsion *J*_2_ are always positive, whereas the exciton binding *K*_2_ is generally negative. Furthermore, we note that in the presented LRC-ωPBEh computations *XC*_2_ is always negative (but this varies with different functionals). We now plot these in a bar graph by stacking the positive contributions on top of each other and using the negative contributions as an offset. Using this representation, the total excitation energy amounts to the top of the left bar for each state. This analysis applied to **ACRFLCN** is presented in Figure 4; the full raw data are provided in Table S1 of the SI. The green bar in Figure 4 represents *h*′ and on top of it, if applicable, we plot *J*_2_ (blue). These terms are offset by the negative contributions *K*_2_ and *XC*_2_ shown on the right. Note that a part of *h*′ is shown below zero and the other above zero as determined by the offset; the total value of *h*′ corresponds to the overall length of the bar.

It is apparent that the *h*′ term (green) for the ^3^LE state of **ACRFLCN** (7.960 eV) is significantly larger than the analogous term for the ^3^CT state (6.041 eV) in line with the latter representing the HOMO/LUMO transition. Nonetheless, ^3^LE is the lowest state due to its enhanced *K*_2_ (and in part *XC*_2_) term. The difference in character between ^3^LE and ^3^CT is also reflected in the exciton size (*d*_*exc*_), which is below 4.0 Å for the former and above 5.5 Å for the latter. More generally, we find an inverse correlation between the *K*_2_ and *d*_*exc*_ values. Moving from ^3^CT to ^1^CT, we find that both states are almost indistinguishable in terms of their energy contributions as well as exciton size. In line with standard models,^18^ the exchange repulsion vanishes for a state with complete charge separation, and this is reflected by an almost vanishing *J*_2_ term of 0.010 eV.

As opposed to the ^1^CT state, the ^1^LE state has a distinctly different appearance when compared to its triplet counterpart showing enhanced exciton size *d*_*exc*_ (4.539 vs 3.803 Å) and less negative *K*_2_ (−3.479 vs −4.204 eV). The obtained exchange repulsion (*J*_2_) is 0.426 eV. If the ^3^LE and ^1^LE wave functions were equivalent aside from the spin-coupling, then their energy difference would be entirely determined by *J*_2_. However, this is clearly not the case here. The gap between ^3^LE and ^1^LE is 1.228 eV; out of these, only +0.426 eV are due to *J*_2_, whereas differences in the Coulomb attraction between the two states (Δ*K*_2_ = 0.725 eV) nominally make a bigger contribution. Changes with the one-electron (*Δh*′ = −0.262 eV) and exchange-correlation (*ΔXC*_2_ = 0.353 eV) terms cancel out approximately. To understand this discrepancy, we compute the formal *J*_2_ term obtained for the singlet state with exactly the same response vector as the ^3^LE state and only the spin coupling reversed. Note that this formal *J*_2_ term does not contribute to the energy and is only regarded for illustrative purposes here. Strikingly, we obtain a formal *J*_2_ term of 2.784 eV (more than twice the gap between ^1^LE and ^3^LE) putting the excitation energy of the associated singlet state to 5.814 eV. We can now interpret the change in the ^1^LE wave functions compared to ^3^LE as an “attempt” of the ^1^LE state to lower its energy by minimizing the exchange repulsion. More accurately, by virtue of being higher in energy, the singlet HOMO/LUMO configuration interacts more strongly with other electronic configurations. This is discussed in more detail below.

**Figure 5:**
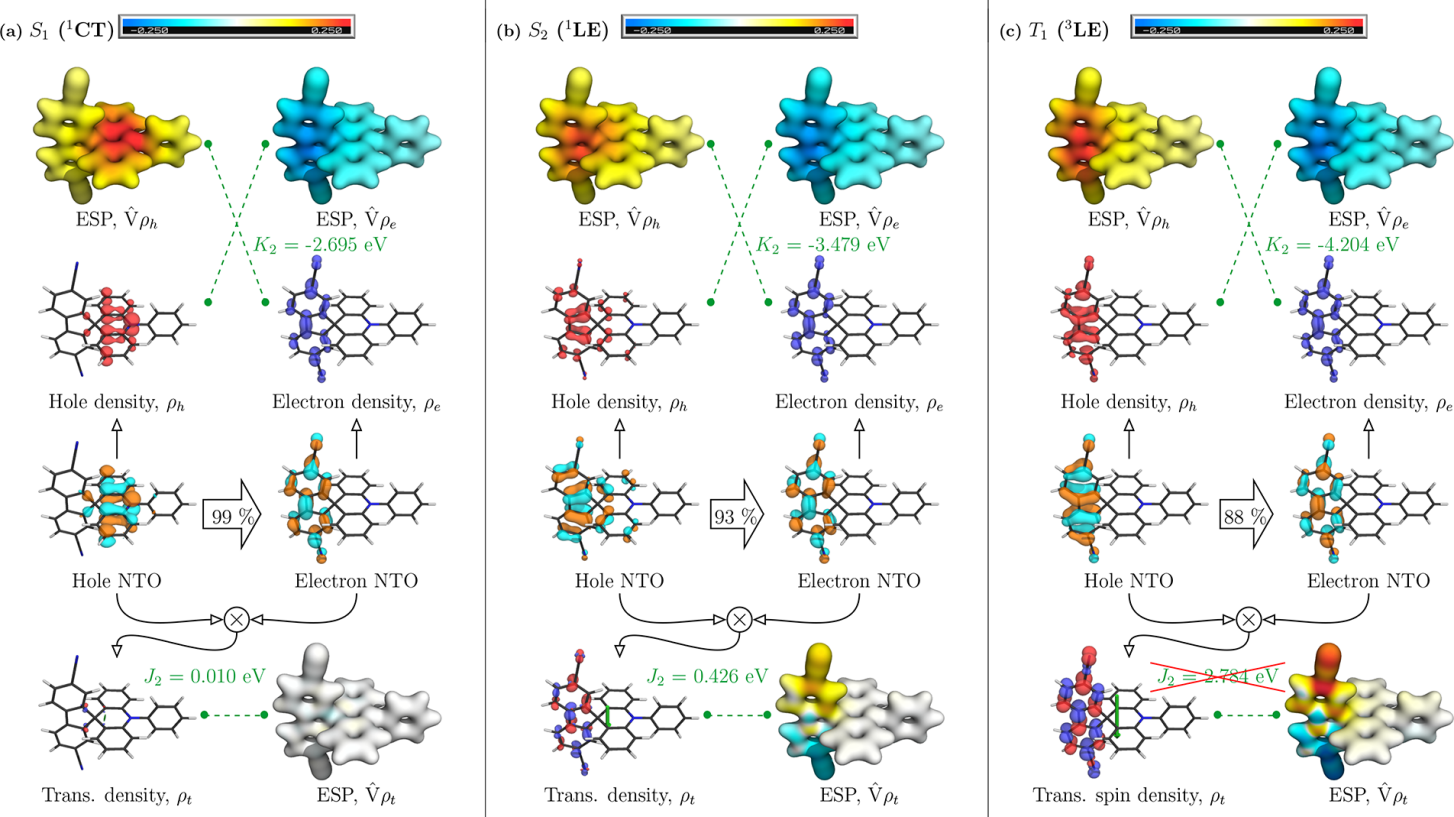
Analysis of the energetic contributions to the (a) *S*_1_ (^1^CT), (b) *S*_2_ (^1^LE), and (c) *T*_1_ (^3^LE) states of **ACRFLCN**. The electron and hole densities and the ESPs they induce are shown at the top. The contributions of the dominant natural transition orbitals are shown in the center. The transition density and its ESP are shown at the bottom. Isovalues used: 0.05 for orbitals, 0.004 for densities, 0.05 for the ESP map.

To provide a graphical interpretation of the two-electron terms, we use a formalism developed in ref (24). This is exemplified for the *S*_1_, *S*_2_, and *T*_1_ states in Figure 5. The *T*_2_, which closely resembles *S*_1_, is presented in the SI (Figure S5). The *hole* and *electron* NTOs (cyan and orange) are shown at the center. The associated *electron* (blue) and *hole* (red) densities and their ESPs, related to *K*_2_, are shown above. Transition densities and their ESPs giving rise to *J*_2_ are shown below.

Starting with the top of Figure 5, we first regard the *hole* and *electron* densities. These are formed as weighted sums over the NTOs, and since only one NTO pair contributes significantly to either state, these densities closely reflect the corresponding NTOs. Considering that only one NTO pair contributes, it is appropriate to apply eq 14; i.e., the *K*_2_ term can be interpreted as a static attraction between the *electron* and *hole* densities. To visualize this term, we compute the electrostatic potentials (ESP) induced by the *electron* and *hole* densities. The *electron* density and its ESP possess a very similar shape for all three states shown, being concentrated on the acceptor group. However, a clear difference is observed for the *hole* density, which is located on the donor group for *S*_1_ but on the acceptor group for the other states. Crucially, the difference is strong enough to produce a markedly different appearance of the ESP of the *hole* density (denoted *Vρ̂*_*h*_ in Figure 5). The ESP has its most positive contribution (red) on the donor for *S*_1_ with a notable decrease on the acceptor group and vice versa for the CT states. The approximate *K*_2_ term is now obtained as the term ⟨ρ_*e*_|*V̂*|ρ_*h*_⟩, that is, by computing the overlap of the *hole* ESP with the *electron* density or vice versa. Clearly ρ_*e*_ lies in an area of lower *hole* ESP for the *S*_1_ state than it does for the other states, and this explains the reduced *K*_2_ term.

Proceeding to the bottom part of Figure 5, we can now discuss the emergence of the *J*_2_ term via the transition density. The product of the hole and electron NTOs leads to the transition density for the excited state (following eq 9 and setting *r*_*e*_ = *r*_*h*_). The computed transition density is shown in blue and red at the bottom of Figure 5. Following eq 8, the *J*_2_ term is given as the self-repulsion of the transition density. In analogy to the Coulomb term, we visualize it via its ESP, and the *J*_2_ term is indicated via the green dotted lines in the lower half of the figure. For the ^1^CT (*S*_1_) state, we find that the transition density and its ESP almost vanish completely everywhere in space, explaining its small *J*_2_ term. Conversely, notable contributions for the transition density are found for the ^1^LE (*S*_2_) state. The transition density induces a strong ESP with a clear dipolar shape, explaining the stronger *J*_2_ term. Note that the dipolar form of the transition density also induces the strong transition dipole moment, shown as a green arrow in Figure 5.

Finally, we revisit the question of the differences in wave functions between the ^1^LE (*S*_2_) and ^3^LE (*T*_1_) states. Both of these are formally composed of the HOMO–1/LUMO transition. However, clear differences in wave functions are apparent in Figure 5. Most notably, the *hole* NTO for the *T*_1_ state (Figure 5 (c)) is entirely localized on the acceptor unit, whereas a notable admixture of donor contributions is found for *S*_2_. This difference leads to enhanced charge transfer as seen from the enhanced *d*_*exc*_ value of *S*_2_ vs *T*_1_ in Figure 4. Importantly, this causes reduced electron–hole overlap and hence a diminished transition density and ESP causing the reduction in the *J*_2_ term. In addition, the delocalization of the hole also leads to more delocalization of its ESP *Vρ̂*_*h*_ toward the donor and hence the reduced *K*_2_ term. A more detailed depiction of the natural transition orbitals involved is shown in Figures S1 and S4. Without going into too much detail here, we want to stress that the NTOs are clearly different between singlet and triplet states in terms of their shapes and occupations, highlighting again the differences in the underlying wave functions. In summary, the *S*_2_ state is differentiated from the *T*_1_ state through an enhanced charge transfer character, which reduces exchange repulsion (via *J*_2_) but also diminishes exciton binding effects (via *K*_2_). Similar differences between singlets and triplets were also observed in related push–pull systems.^61^

An alternative viewpoint on these phenomena has been presented in ref (62), highlighting that due to correlation the singlet–triplet gaps are often only about half the value that would be expected from the HOMO/LUMO exchange integral. Furthermore, it is interesting to view these results in the context of unrestricted (UKS) and restricted open-shell (ROKS) Kohn–Sham computations on singlet excited states.^63−65^ A spin-broken UKS state only experiences half the *J*_2_ term compared to the spin-adapted singlet of the TOTEM shown in eq 5, meaning that UKS should severely underestimate singlet state energies. However, we have just shown that correlation and relaxation effects also lead to halving of the *J*_2_ term meaning that, in summary, spin-broken UKS is a surprisingly good approximation to singlet excited states.

In summary, we find that the locally excited triplet state of **ACRFLCN** lies below the HOMO/LUMO transition (^3^*CT*) due to its enhanced Coulomb binding term (*K*_2_). By contrast, the exchange repulsion (*J*_2_) dominates for the singlets, keeping the singlet HOMO/LUMO transition (^1^*CT*) the lowest singlet state.

### 3.2 Naphthalene: Ionic and Covalent States

Naphthalene is a paradigmatic case of an aromatic molecule, possessing a number of rather closely spaced ππ* states with distinct characteristics despite being all constructed from the same set of MOs. Indeed, discussion of the MOs alone is by far not sufficient to characterize these states, and a number of different viewpoints, such as Platt’s nomenclature^66^ and Pariser’s +/– nomenclature,^30^ have been introduced to obtain a clearer picture.^36^ The critical realization in this case is that not only the MO transitions as such are of relevance but that also the relative signs between the different interacting configurations are decisive.^17,27,28^ The effect of this is seen in physically observable quantities such as oscillator strengths and energies but also resurfaces in the computational description posing severe and sometimes unexpected challenges.^32,33^

**Figure 6:**
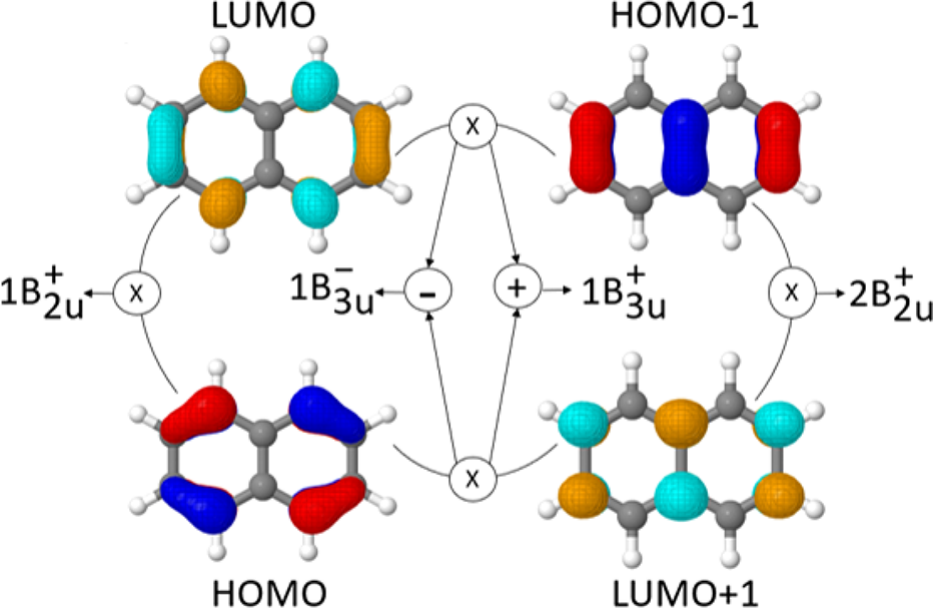
Linear combinations of canonical orbitals leading to the first four singlet and triplet excited states of naphthalene according to the dominant configuration.

In the following, we analyze the lowest four singlet and triplet excited states of naphthalene. All these states can be explained by transitions from the HOMO–1 and HOMO to the LUMO and LUMO+1, which are presented graphically in Figure 6. The HOMO/LUMO and HOMO–1/LUMO+1 transitions mix only weakly and yield two individual states of *B*_2*u*_ symmetry, denoted 1*B*_2*u*_^+^ and 2*B*_2*u*_^+^, as shown to the left and right. Conversely, strong mixing is observed between the HOMO/LUMO+1 and HOMO–1/LUMO transitions yielding two states (1*B*_3*u*_^–^ and 1*B*_3*u*_^+^) distinguished only by the sign used in their linear combination. Note that the signs in the linear combination depend on the orbital signs, and we use the conventions introduced by Pariser.^30^ Furthermore, it is important to realize that the singlet “+” states are interpreted as ionic states and the singlet “–” states as covalent states within valence-bond theory.^31^ The four possible states formed are shown in the middle line of Figure 6; coupling them with singlet or triplet spin yields the eight states of interest.

Computational data on the states are presented in Table 3. Here, we present excitation energies, oscillator strengths, and configurations along with the LOC descriptor (see eq 17). The LOC descriptor provides a convenient means to differentiate between “+” and “–” states where only the former possess nonvanishing LOC values. The lowest excited state is 1^3^*B*_2*u*_^+^ (or ^3^*L*_*a*_) at 3.071 eV, reached via the HOMO/LUMO transition. Three more triplet states (1^3^*B*_3*u*_^+^, 1^3^*B*_3*u*_^–^, and 2^3^*B*_2*u*_^+^) follow in the range between 4.212 and 4.684 eV. The first singlet state (1^1^*B*_3*u*_^–^, also known as ^1^*L*_*b*_) is dark and lies at 4.779 eV. Interestingly, *S*_1_ possesses the same character as *T*_3_ highlighting the different energetic contributions that act on singlet and triplet states leading to different ordering of the states in the respective spin manifolds. The second singlet state (1^1^*B*_2*u*_^+^ or ^1^*L*_*a*_), finally, is reached via the HOMO/LUMO transition in analogy to *T*_1_. With an oscillator strength of 0.097, it forms the first notable band in the absorption spectrum of naphthalene. Two more bright states (1^1^*B*_3*u*_^+^ and 2^1^*B*_2*u*_^+^) follow near 7 eV. The state ordering, i.e., the dark ^1^*L*_*b*_ state below the bright HOMO/LUMO transition (^1^*L*_*a*_) followed by even brighter high-energy states, is well documented in the literature,^27,67^ and we explain this energetic ordering in the following.

**Table 3:** Excitation Energies (Δ*E*, eV), Oscillator Strengths (f), Leading Configurations, and LOC Descriptor Computed for the First Four Singlet and Triplet States of Naphthalene at the LRC-ωPBEh/def2-SV(P) (TDA) Level of Theory

State	Δ*E*	f	Configurations^a^	LOC
1^3^*B*_2*u*_^+^ (*T*_1_)	3.071	–	0.93*hl*	0.138
1^3^*B*_3*u*_^+^ (*T*_2_)	4.212	–	0.74*h*_1_*l* + 0.64*hl*_1_	0.063
1^3^*B*_3*u*_^–^ (*T*_3_)	4.510	–	0.74*hl*_1_*-* 0.65*h*_1_*l*	0.000
2^3^*B*_2*u*_^+^ (*T*_4_)	4.684	–	0.95*h*_1_*l*_1_	0.088
1^1^*B*_3*u*_^–^ (*S*_1_)	4.779	0.000	0.70*hl*_1_*-* 0.70*h*_1_*l*	0.000
1^1^*B*_2*u*_^+^ (*S*_2_)	5.085	0.097	0.93*hl*	0.074
1^1^*B*_3*u*_^+^ (*S*_3_)	6.815	2.023	0.68*h*_1_*l* + 0.68*hl*_1_	0.040
2^1^*B*_2*u*_^+^ (*S*_4_)	6.990	0.393	0.89*h*_1_*l*_1_	0.072

a Dominant configurations and coefficients; *h*_*x*_*l*_*y*_ refers to the HOMO-*x*/LUMO+*y* transition.

**Figure 7:**
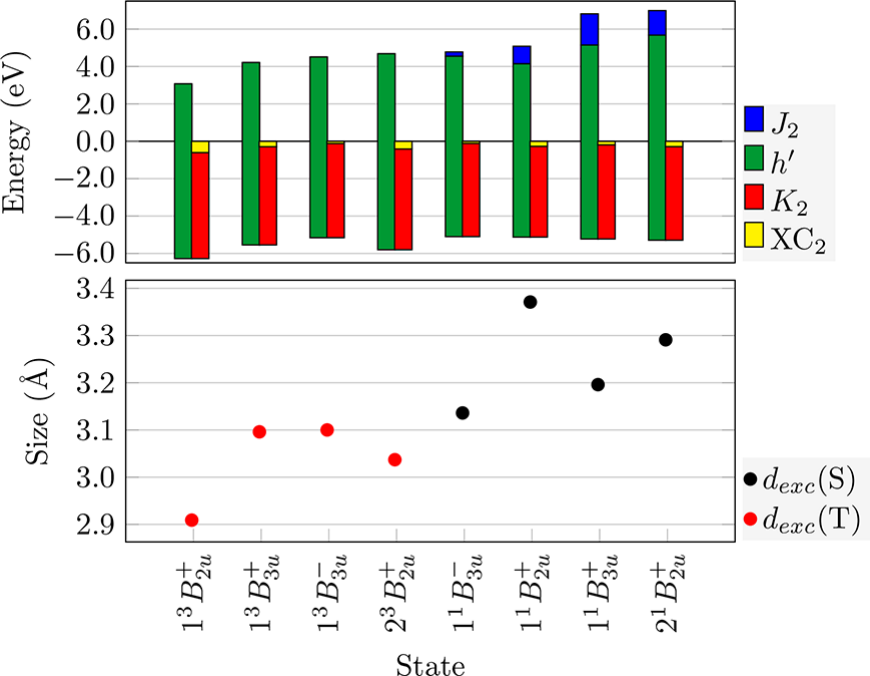
Energy components and exciton sizes for the computed excited states of naphthalene. Exciton sizes *d*_*exc*_ are plotted with singlets in black and triplets in red.

Table 3 illustrates the importance of post-MO contributions in the analysis of excitation energies. The states presented span a range of almost 4 eV despite being composed of the same four MOs. It is particularly noteworthy that *T*_1_ is separated from all the other states by more than 1 eV, while *S*_3_ and *S*_4_ are separated from the others by almost 2 eV. Strikingly, there is a difference of more than 2.5 eV between the 1^3^*B*_3*u*_^+^ and 1^1^*B*_3*u*_^+^ states, which are constructed via analogous orbital transitions and only differ in electron spin. We now endeavor to explain these differences using the energy component analysis for naphthalene, as presented in Figure 7.

Unlike **ACRFLCN**, all of the excited states in naphthalene can be considered locally excited as seen by their low exciton sizes (*d*_*exc*_ < 3.5 Å). As a consequence, all *K*_2_ values are fairly similar (around −5 eV) with no dramatic changes as seen in Figure 4. Nonetheless, the balance between the different terms has a crucial effect. We first investigate why *T*_1_ (1^3^*B*_2*u*_^+^) lies more than 1 eV below *T*_2_ (1^3^*B*_3*u*_^+^). Only 0.413 eV are due to the orbital energies (Δ*h*′). Additional important contributions come from Coulomb binding (Δ*K*_2_ = 0.414 eV) and the response of the exchange-correlation potential (Δ*XC*_2_ = 0.313 eV). These indicate a more local nature for *T*_1_, which is also reflected in its smaller exciton size (*d*_*exc*_ = 2.909 Å) and higher LOC value.

Next, we may ask why the 1^3^*B*_3*u*_^–^ state lies above the 1^3^*B*_3*u*_^+^ state by about 0.3 eV. Being composed of the same orbital transitions, both states have almost equivalent one-electron components (Δ*h*′ = −0.088 eV). Conversely, the difference lies in exciton binding (Δ*K*_2_ = 0.209 eV) and exchange-correlation (Δ*XC*_2_ = 0.178 eV). These values are enhanced due to the more local nature of the 1^3^*B*_3*u*_^+^ state as indicated by the *LOC* descriptor in Table 3. The next state, 2^3^*B*_2*u*_^+^, is the HOMO–1/LUMO+1 transition. It lies above the 1^3^*B*_3*u*_^–^ state on account of the increased one electron component (Δ*h*′ = 0.816 eV), consistent with the fact that the canonical orbitals involved (HOMO–1/LUMO+1) are further apart energetically. This increase is offset only in part by the two-electron terms.

The first singlet state (*S*_1_, 1^1^*B*_3*u*_^–^) is the counterpart to the *T*_3_ state. We find that their energy components are almost identical, the only major difference being the *J*_2_ term of 0.234 eV, which is responsible for the energetic difference between the two states.

The HOMO/LUMO singlet state (1^1^*B*_2*u*_^+^) follows as *S*_2_, being the sixth excited state overall. Its one-electron component of *h*′ = 9.270 eV is lower than the four preceding states in line with being the HOMO/LUMO state. However, it is pushed up in energy due to its exchange repulsion term of *J*_2_ = 0.939 eV. In addition, when compared to the corresponding triplet state (*T*_1_), we find that binding via *K*_2_ and *XC*_2_ is strongly reduced. Indeed, the gap between *S*_2_ and *T*_1_ is 2.014 eV, meaning that only half of this can be explained via the *J*_2_ term where the other two-electron terms account for the rest. Conversely, as described above, we can evaluate the formal *J*_2_ term obtained for a singlet state with exactly the same response vector as *T*_1_. We obtain a formal *J*_2_ value of 4.203 eV and are left with the same result seen for the ^1^LE and ^3^LE states of **ACRFLCN**: the formal *J*_2_ term is strongly reduced when going from the triplet to the singlet, but this is done at the cost of other two-electron terms.

Next, the 1^1^*B*_3*u*_^+^ state is found as *S*_3_ lying 1.730 eV higher than the previous states. This state is dominated by the HOMO–1/LUMO and HOMO/LUMO+1 transitions just as *T*_2_, *T*_3_, and *S*_1_. However, its energy components differ markedly from these states. The one-electron components (*h*′ = 10.373 eV) are significantly higher, and a prominent exchange term (*J*_2_ = 1.672 eV) is observed, both of which contribute to the higher excitation energy. Finally, the 2^1^*B*_2*u*_^+^ state is obtained as *S*_4_ possessing an enhanced one-electron gap (*h*′ = 10.971 eV) due to being the HOMO–1/LUMO+1 transition along with a substantial exchange repulsion (*J*_2_ = 1.313 eV).

**Figure 8:**
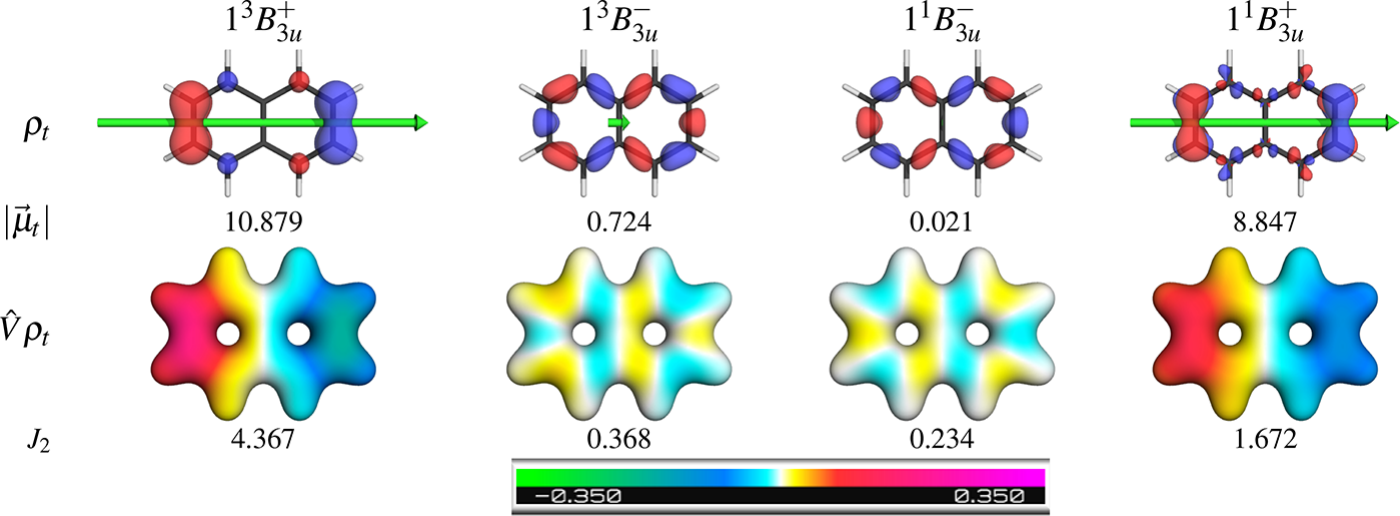
Transition densities (ρ_*t*_), transition dipole moment lengths (|μ⃗_*t*_|, Debye), ESPs (*Vρ̂*_*t*_), and computed exchange repulsion components (*J*_2_, eV) for the *B*_3*u*_ states of naphthalene. The ESP color coding is shown at the bottom. The transition dipole moment (μ⃗_*t*_) is shown at the top as a green arrow of length 2 μ⃗_0*I*_/*e*. Isovalues used: 0.004 for densities, 0.05 for the ESP map.

To understand the observed trends in some more detail, we plot the transition densities and their ESPs, in analogy to the bottom part of Figure 5. These are shown in Figure 8 for the *B*_3*u*_ states all of which are even mixtures of the *h*_1_*l* and *hl*_1_ configurations, only differentiated by the relative signs. Analogous plots for the *B*_2*u*_ states are presented in Figure S14. First, we observe that the transition densities of all the “+” states are concentrated on the atoms, whereas they are between them for the “–” states.^29,36^ This is in agreement with the LOC descriptors: The “+” states show enhanced LOC and hence *electron* and *hole* are on the same basis function on a given atom, producing transition density on that atom. For the “–” states, on the other hand, the transition density is only formed via “differential overlap” terms, i.e., as the overlap of basis functions on different atoms (see also ref (68)). As a consequence, the transition moments and ESPs are significantly reduced for the “–” states when compared to the “+” states, which is reflected in the oscillator strengths and energies of the singlet states shown in Table 3. We find a general positive correlation between *J*_2_ and the transition moments explaining why brighter states are pushed up in energy (cf. Table 3).

Finally, we note the differences in the shape of the transition density for the singlet and triplet *B*_3*u*_^+^ states. The triplet state only shows π contributions on the outer atom. Interestingly, for the singlet, there are also some σ contributions on all the atoms involved. These σ contributions serve in lowering the *J*_2_ term,^24^ albeit at the cost of raising *h*′ and *K*_2_ as discussed above. These σ contributions are also apparent in the NTO representation (Figures S12, S13), where they appear as σσ* excitations with collective weights of a few percent.

In summary, we note that the presented analysis scheme provided a rationale for the energy ordering of all eight states considered. In particular, we could explain why the HOMO/LUMO transition does not form the lowest singlet state by considering its enhanced *J*_2_ term. The discussion also showed that the wave functions involved are more complicated than one might presume at first. In particular, mixing of different configurations is involved lowering the *J*_2_ terms of the ionic singlet states. This discussion also highlights why the computational description of the related ionic and covalent states can be highly challenging and, specifically, why correlation of the σ system is decisive to obtain accurate energies.^48−51^

## 4 Conclusions and Outlook

Within this work, we presented an energy component analysis for excited states, elucidated the physical meaning of the different terms, and used it to answer one perplexing question: why is the lowest excited state of a given spin multiplicity not always derived from the HOMO/LUMO transition? It was shown that excited state energies can be understood as a sum of a weighted orbital energy difference (*h*′), a Coulomb attraction term (*K*_2_), an exchange repulsion term (*J*_2_), and additional two-electron exchange/correlation terms (*XC*_2_). The Coulomb attraction term favors locally excited states, in particular triplets. The exchange repulsion term, on the other hand, disfavors locally excited singlet states. Therefore, two cases can be constructed where the HOMO/LUMO transition is not the lowest state: (i) If the HOMO/LUMO transition is of CT character, then a locally excited triplet may be of lower energy. (ii) If the HOMO/LUMO transition is locally excited, then a different singlet state may be lower by minimizing exchange repulsion (either through its CT character or interaction of different electronic configurations). To exemplify case (i), we used the push–pull system **ACRFLCN** while the naphthalene molecule was used for case (ii).

For **ACRFLCN**, we found indeed that the HOMO/LUMO transition formed the *T*_2_ state. This was explained by the fact that the HOMO–1/LUMO transition represented a locally excited state with enhanced Coulomb attraction, which pushed it below *T*_2_ despite its larger MO energy gap. The order was reversed for the singlets due to enhanced exchange repulsion in the locally excited state, compensating for Coulomb attraction.

It was found that for naphthalene the *T*_1_ state is reached via the HOMO/LUMO transition, whereas *S*_1_ is reached by a linear combination of the HOMO–1/LUMO and HOMO/LUMO+1 transitions forming the *L*_*b*_/1^1^*B*_3*u*_^–^ state. The eight computed excited states for naphthalene were all found to be reached via some linear combination of transitions which involve only four canonical orbitals: HOMO–1, HOMO, LUMO, and LUMO+1. We have shown how the presented energy component analysis scheme offers a rationale for the ordering of all these states.

In a more general sense, we have found in agreement with previous work^62^ that the simple picture of equating the singlet–triplet gap to twice the HOMO/LUMO exchange integral is not quantitatively accurate. Evaluating the formal exchange repulsion using the triplet states yielded about twice the singlet–triplet gap actually obtained. Conversely, the singlet states lower their energy via additional orbital relaxation and correlation terms redistributing the energy penalty from *J*_2_ into *h*′ and *K*_2_ terms. These observations provide new insight into the challenges of providing a balanced computational description for molecular singlet and triplet states of ionic or covalent character, which have been observed for single^33,69,70^ and multireference methods.^48−50^ A deeper understanding of these issues could ultimately help in the development of targeted corrections to these methods.

We have presented our analysis protocol for TDDFT being a convenient and widely used method. However, we want to stress that the general physics involved is independent of the computational protocol. It is certainly also possible to use this type of analysis in a correlated ab initio framework. For example, in the context of the algebraic diagrammatic construction (ADC) method, the *h*′ term could be represented via zeroth order energies, *J*_2_ and *K*_2_ via first order, and *XC*_2_ made up of higher order terms. Moreover, many of the concepts mentioned are naturally included in the GW framework.^45,46^ Alternatively, it is possible to separate the analysis from any quantum chemistry method using Dyson orbitals and many-body ionization potentials/electron affinities as a starting point. Therefore, we hope that the presented analysis will be useful for both practical calculations and formal discussions.

## Supporting Information Available

Numerical values of energy components (Table S1), NTOs and ESP analysis of **ACRFLCN** (Figures S1–S5), NTOs and ESP analysis of naphthalene (Figures S6–S14) (PDF)
