# Supplementary material for: Energy Component Analysis for Electronically Excited States of Molecules: Why the Lowest Excited State Is Not Always the HOMO/LUMO Transition
Source: J Chem Theory Comput. 2023 Apr 6;19(8):2340–52. doi: 10.1021/acs.jctc.3c00125 (PMC10134415; doi:10.1021/acs.jctc.3c00125)
Supplement: Supplementary file 1 — ct3c00125_si_001.pdf [file ct3c00125_si_001.pdf]

Electronic Supporting Information

for

Energy component analysis for electronically excited states of molecules - Why the lowest excited state is not always the HOMO-LUMO transition

Patrick Kimber, Felix Plasser*

Department of Chemistry, Loughborough University, Loughborough, LE11 3TU, U.K.
E-mail: f.plasser@lboro.ac.uk
Phone: +44 1509 226946

Table S1 - Calculated components of the excitation energy for naphthalene and ACRFLCN. H is the contribution from the core Hamiltonian, J_1 and K_1 are the one electron Coulomb and Hartree-Fock exchange energies repsectively, J_2 is the contribution arising from the transition density self-repulsion, K_2 is the Coulomb binding between electron and hole quasiparticles. XC_1 and XC_2 terms are the one and two electron terms respectively from the exchange-correlation functional

State	1-electron					2-electron				Total
	H	J_1	K_1	XC_1	h'	J_2	K_2	XC_2	Sum	
					ACRFLCN					
T_1	10.007	-6.496	4.156	0.023	7.690	2.784	-4.204	-0.456	-4.660	3.030
T_2	97.591	-96.945	4.425	0.970	6.041	0.017	-2.709	-0.008	-2.717	3.324
S_1	98.227	-97.603	4.424	0.979	6.027	0.010	-2.695	-0.006	-2.691	3.336
S_2	54.209	-51.312	4.228	0.303	7.428	0.426	-3.479	-0.103	-3.157	4.271
					Naphthalene					
T_1	5.382	-1.285	5.344	-0.095	9.346	4.203	-5.669	-0.606	-6.275	3.071
T_2	9.899	-5.472	5.347	-0.016	9.759	4.385	-5.255	-0.293	-5.547	4.212
T_3	9.139	-4.828	5.315	0.046	9.671	0.371	-5.046	-0.115	-5.161	4.510
T_4	10.557	-5.254	5.345	-0.161	10.487	4.101	-5.391	-0.413	-5.803	4.684
S_1	10.496	-6.206	5.317	0.041	9.647	0.234	-4.994	-0.108	-4.867	4.780
S_2	11.065	-7.403	5.337	0.272	9.270	0.939	-4.856	-0.271	-4.188	5.082
S_3	15.751	-11.122	5.462	0.282	10.373	1.672	-5.033	-0.195	-3.556	6.817
S_4	16.470	-11.276	5.518	0.258	10.971	1.313	-5.007	-0.287	-3.981	6.990

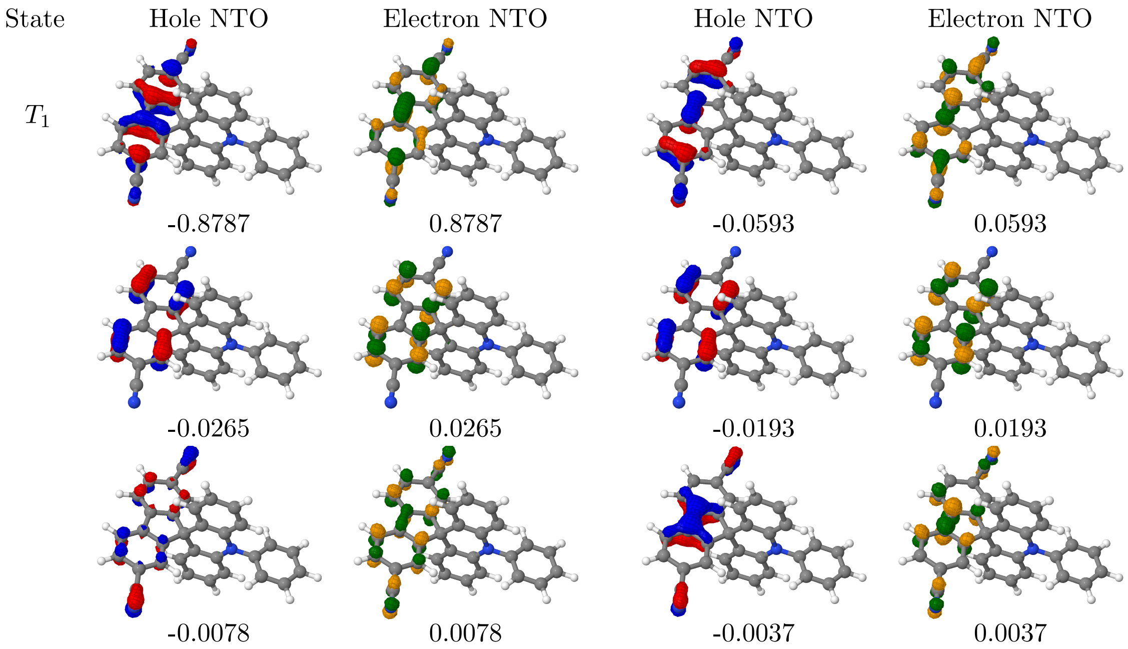

Figure S1 - The natural transition orbital (NTO) pairs for the T_1 state of ACRFLCN, the numerical contribution of each pair is shown underneath the orbitals

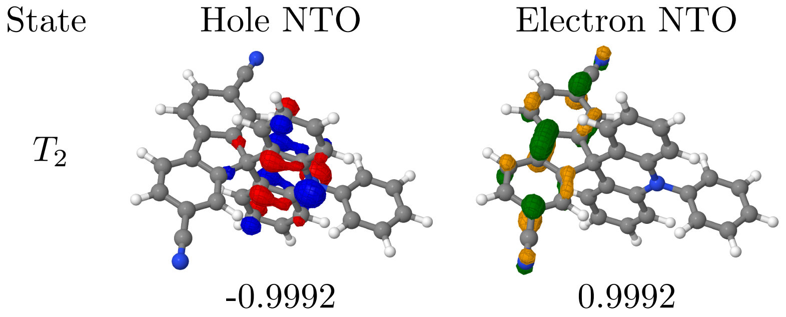

Figure S2 - The natural transition orbital (NTO) pair for the T_2 state of ACRFLCN, the numerical contribution of the pair is shown underneath the orbitals

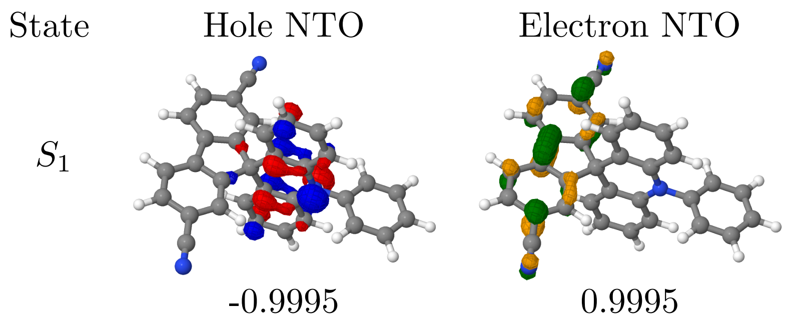

Figure S3 - The natural transition orbital (NTO) pair for the S_1 state of ACRFLCN, the numerical contribution of the pair is shown underneath the orbitals

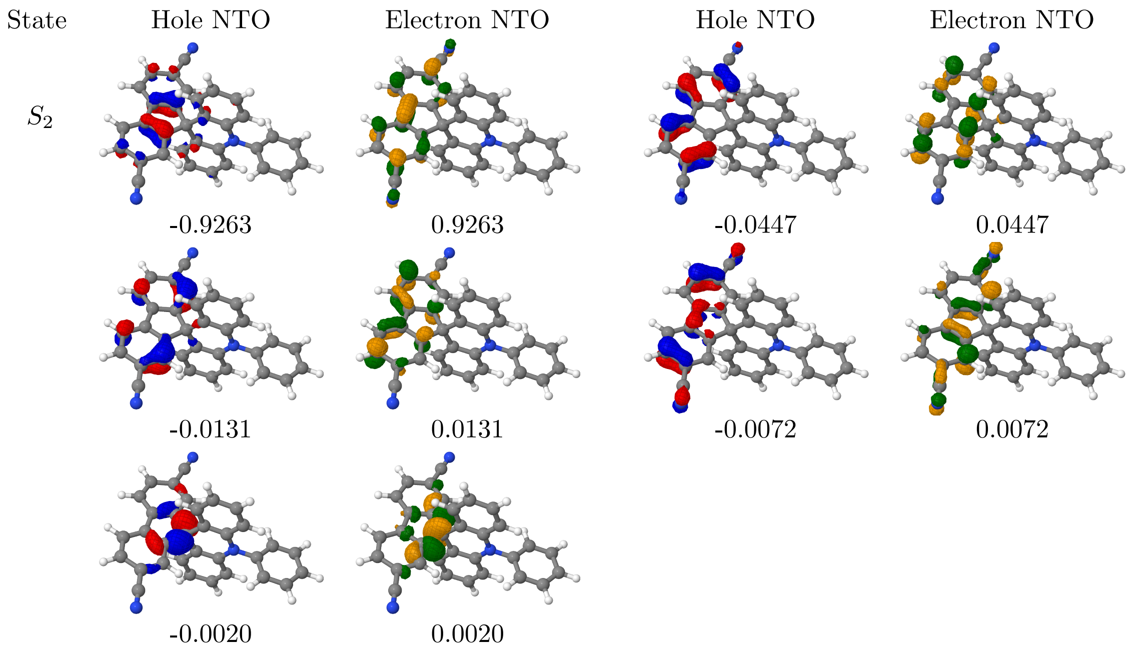

Figure S4 - The natural transition orbital (NTO) pairs for the S_2 state of ACRFLCN, the numerical contribution of each pair is shown underneath the orbitals

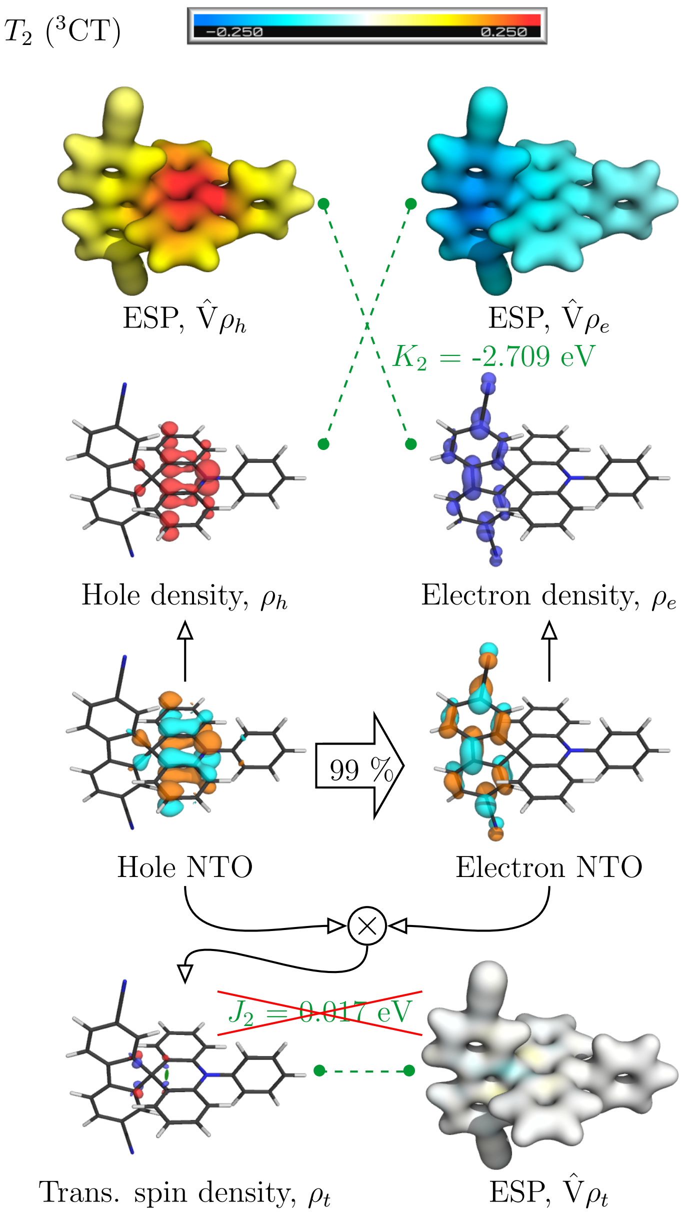

Figure S5 - Analysis of the energetic contributions to the T_2 (^{3}CT) state of ACRFLCN. The electron and hole densities, and the ESPs they induce are shown. The Coulomb interaction between hole/electron density and electron/hole ESP are shown as a green dashed line. The contributions of the dominant natural transition orbitals are shown in the centre. The transition density and its ESP are shown at the bottom. Isovalues used: 0.05 for orbitals, 0.004 for densities, 0.05 for the ESP map.

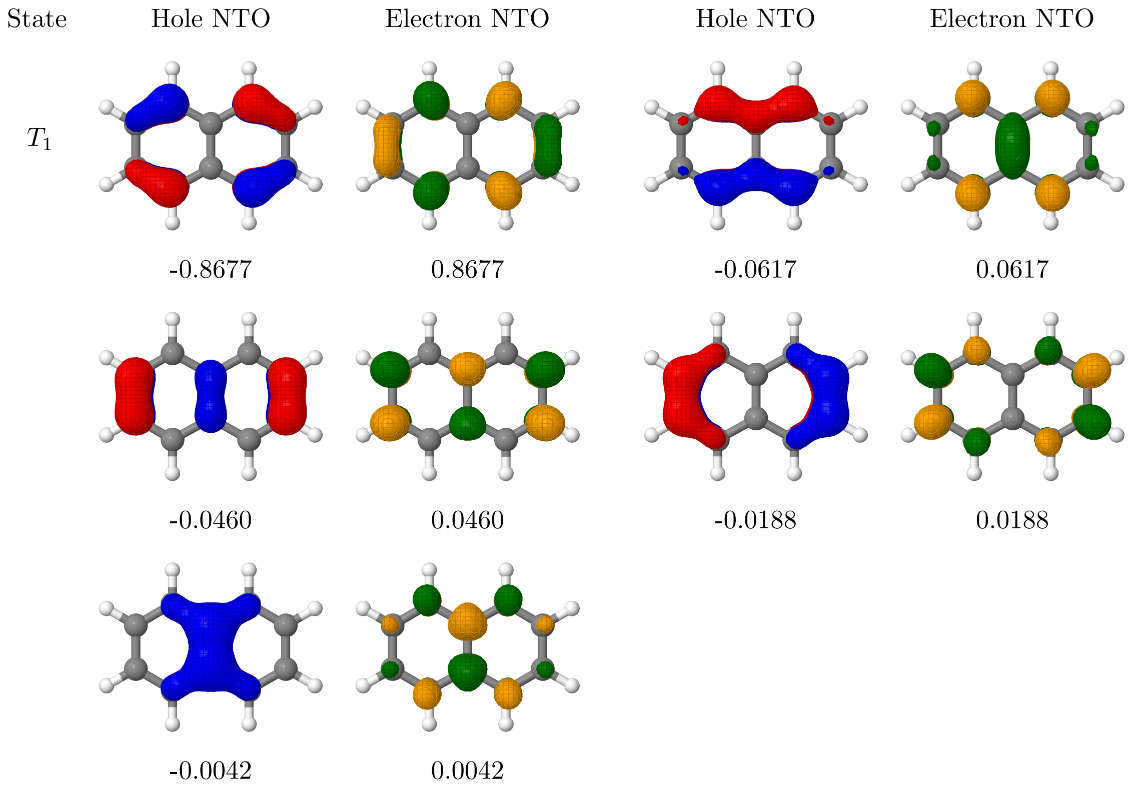

Figure S6 - The natural transition orbital (NTO) pairs for the T_1 state of naphthalene, the numerical contribution of each pair is shown underneath the orbitals

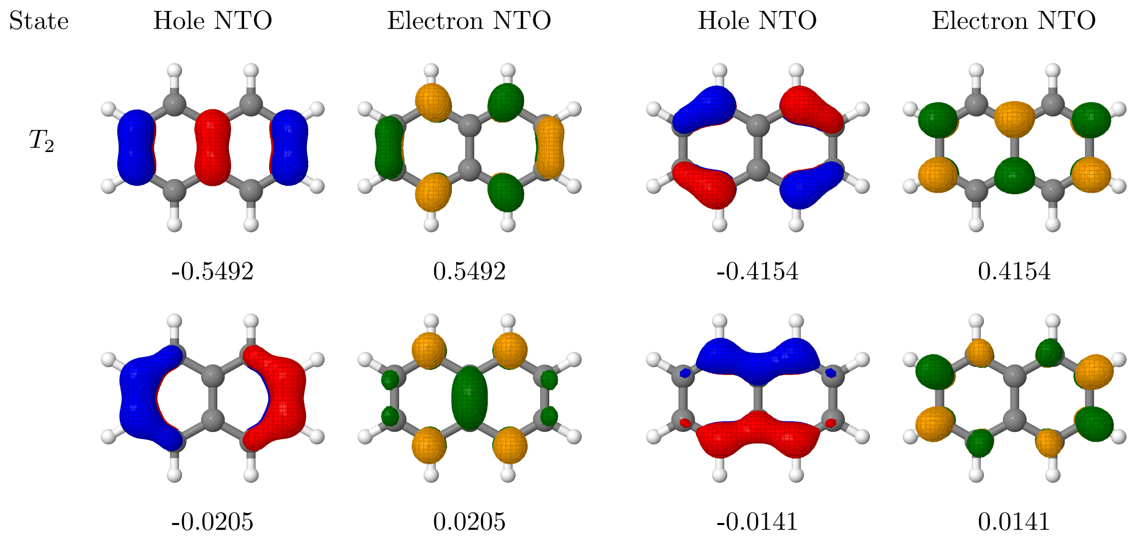

Figure S7 - The natural transition orbital (NTO) pairs for the T_2 state of naphthalene, the numerical contribution of each pair is shown underneath the orbitals

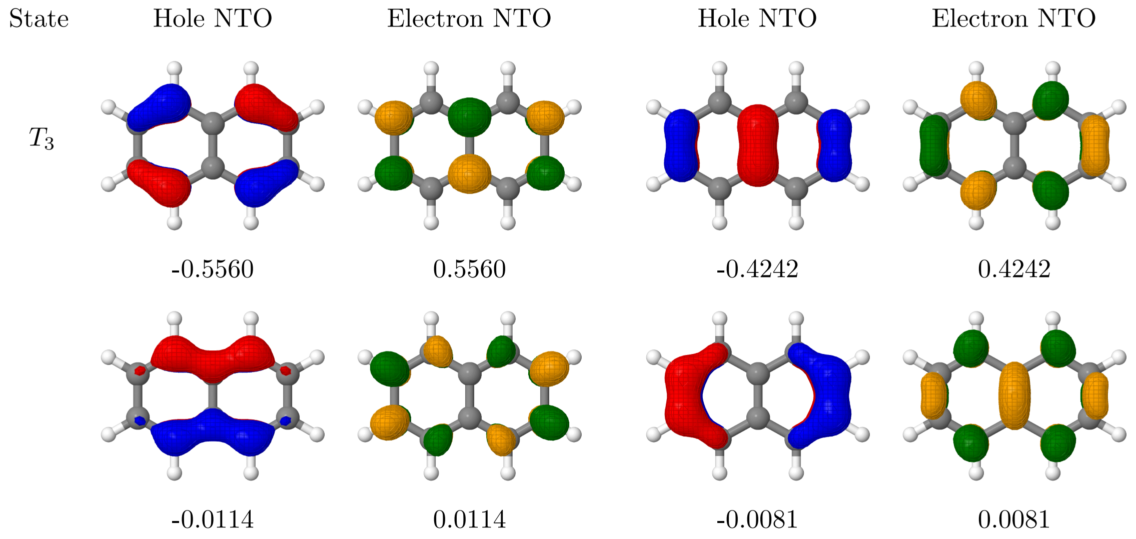

Figure S8 - The natural transition orbital (NTO) pairs for the T_3 state of naphthalene, the numerical contribution of each pair is shown underneath the orbitals

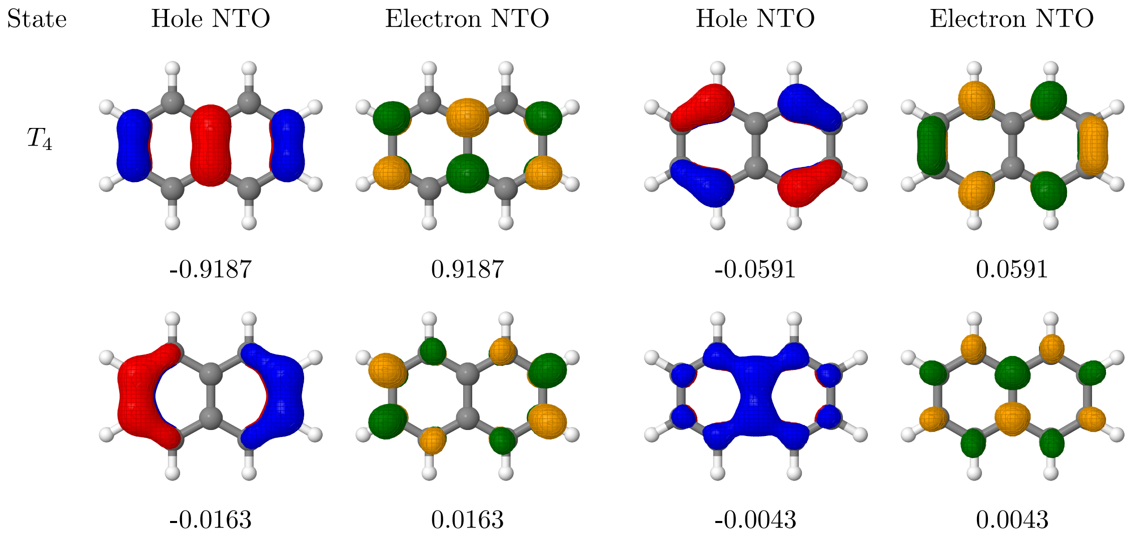

Figure S9 - The natural transition orbital (NTO) pairs for the T_4 state of naphthalene, the numerical contribution of each pair is shown underneath the orbitals

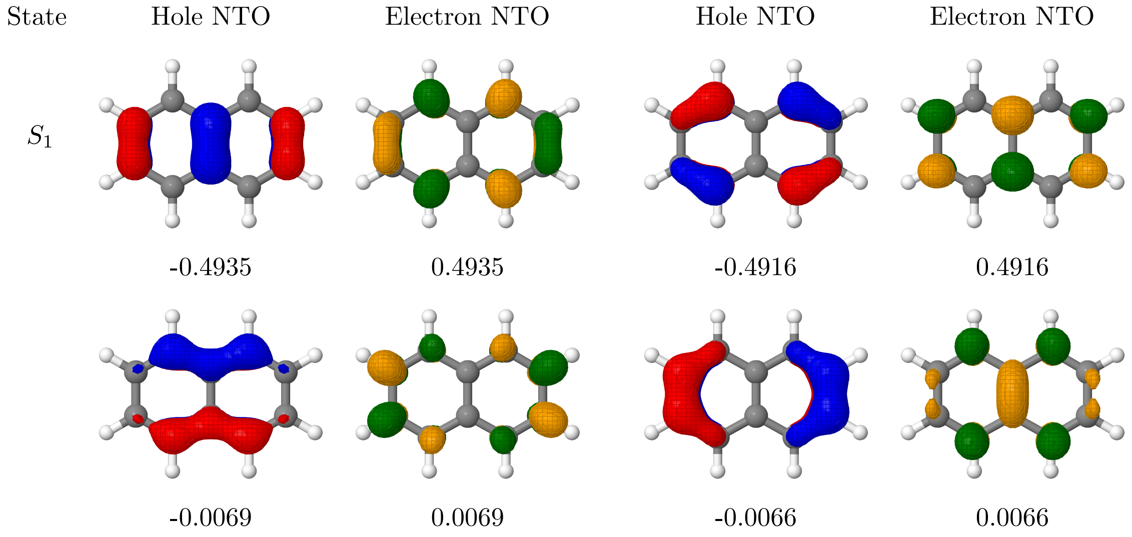

Figure S10 - The natural transition orbital (NTO) pairs for the S_1 state of naphthalene, the numerical contribution of each pair is shown underneath the orbitals

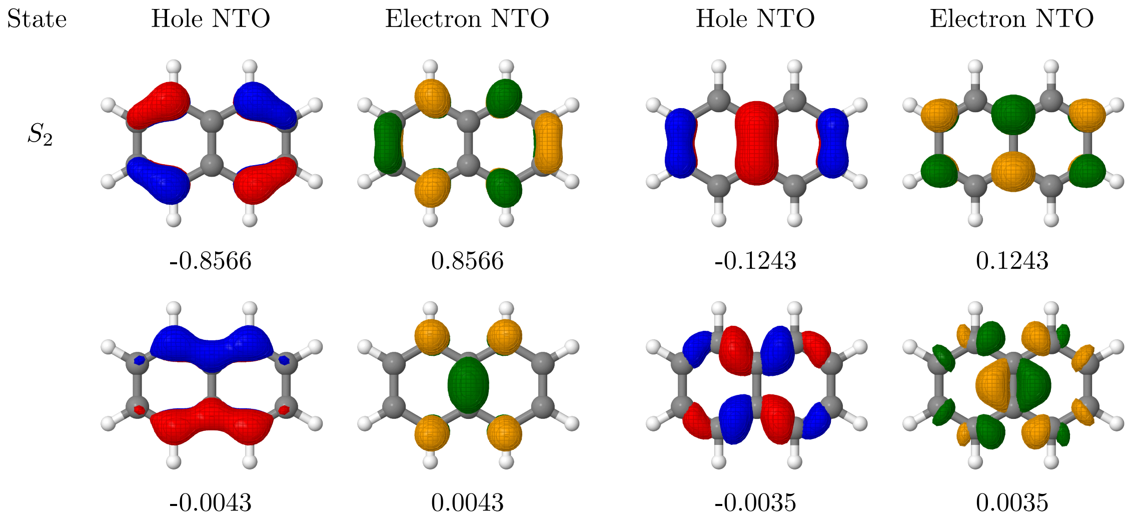

Figure S11 - The natural transition orbital (NTO) pairs for the S_2 state of naphthalene, the numerical contribution of each pair is shown underneath the orbitals

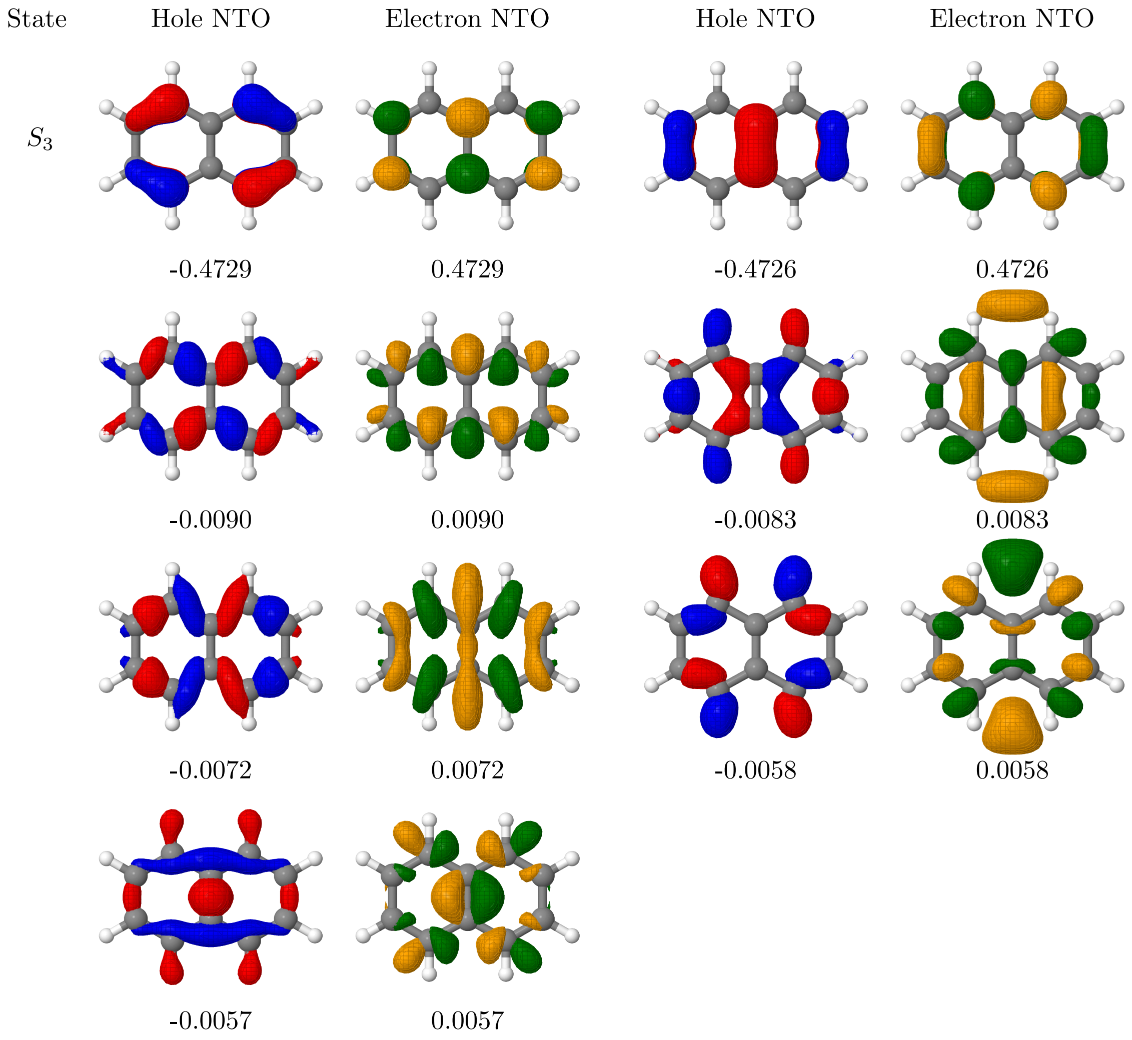

Figure S12 - The natural transition orbital (NTO) pairs for the S_3 state of naphthalene, the numerical contribution of each pair is shown underneath the orbitals

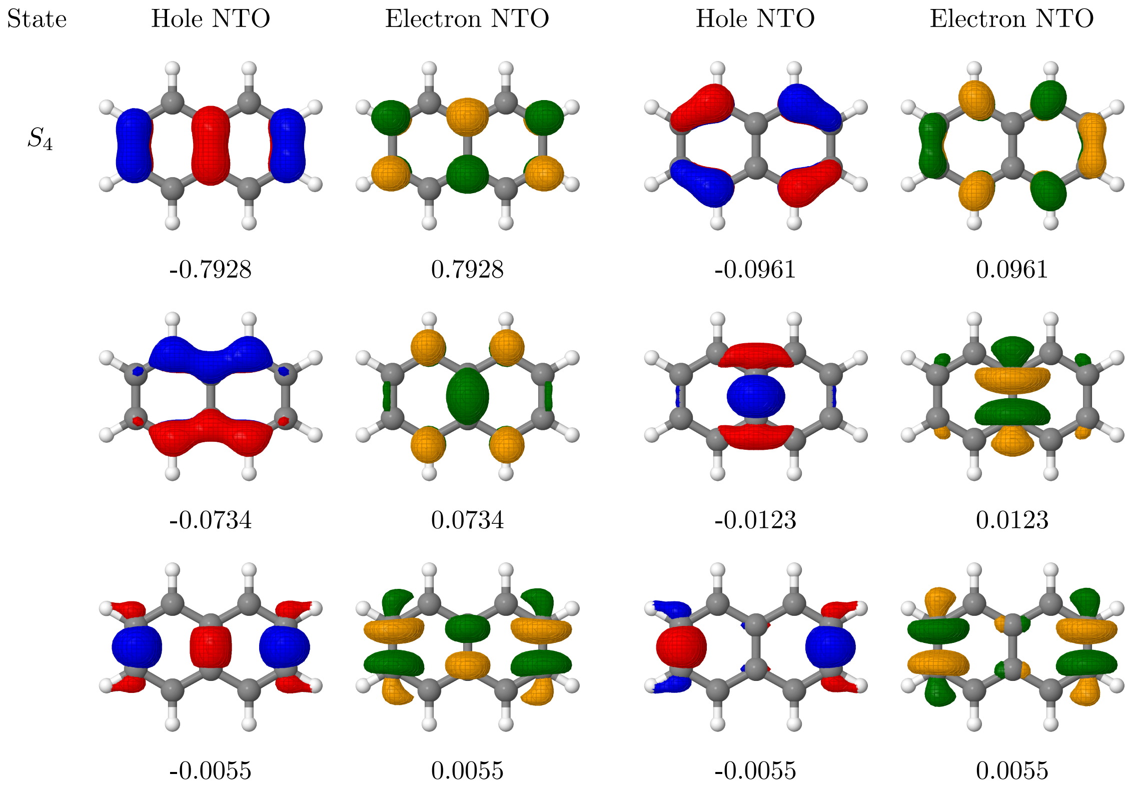

Figure S13 - The natural transition orbital (NTO) pairs for the S_4 state of naphthalene, the numerical contribution of each pair is shown underneath the orbitals

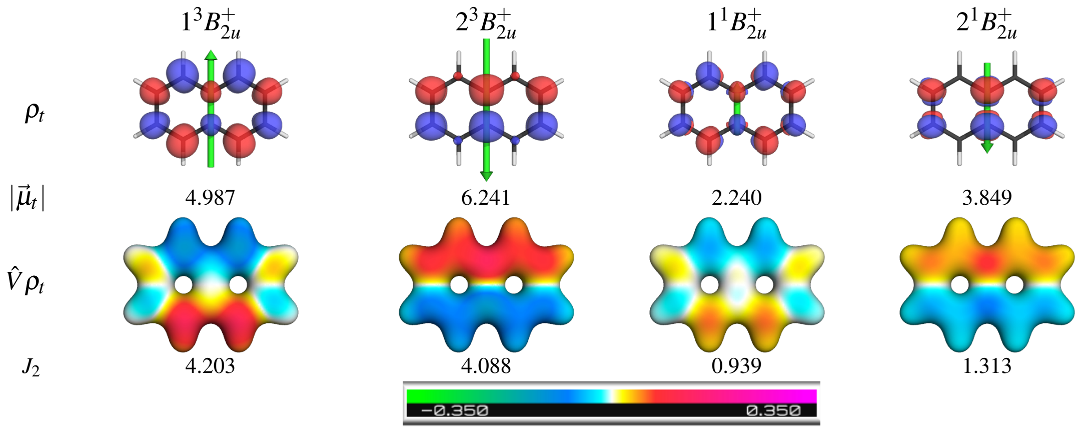

Figure S14 - The transition densities (ρ_t), transition dipole moment lengths ($|\vec{\mu}_t|$, Debye), ESPs ($\hat{V}\rho_t$), and computed exchange repulsion components (J_2) in eV for the B_{2u} states of naphthalene. The ESP colour coding is shown. The transition dipole moment ($\vec{\mu}_t$) is shown as a green arrow of length $2\vec{\mu}_{0I}/e$. Isovalues used: 0.05 for orbitals, 0.004 for densities, 0.05 for the ESP map.
